# Fosamprenavir or atazanavir once daily boosted with ritonavir 100 mg, plus tenofovir/emtricitabine, for the initial treatment of HIV infection: 48-week results of ALERT

**DOI:** 10.1186/1742-6405-5-5

**Published:** 2008-03-28

**Authors:** Kimberly Y Smith, Winkler G Weinberg, Edwin DeJesus, Margaret A Fischl, Qiming Liao, Lisa L Ross, Gary E Pakes, Keith A Pappa, C Tracey Lancaster, the ALERT (COL103952) Study Team

**Affiliations:** 1 Section of Infectious Diseases, Rush University Medical Center, Chicago, Illinois, USA; 2 Infectious Diseases Service, Kaiser Permanente, Atlanta, Georgia, USA; 3 Orlando Immunology Center Research Facility, Orlando Immunology Center, Orlando, Florida, USA; 4 AIDS Clinical Research Unit, University of Miami, Miami, Florida, USA; 5 Infectious Diseases, GlaxoSmithKline, Research Triangle Park, North Carolina, USA

## Abstract

**Background:**

Once-daily (QD) ritonavir 100 mg-boosted fosamprenavir 1400 mg (FPV/r100) or atazanavir 300 mg (ATV/r100), plus tenofovir/emtricitabine (TDF/FTC) 300 mg/200 mg, have not been compared as initial antiretroviral treatment. To address this data gap, we conducted an open-label, multicenter 48-week study (ALERT) in 106 antiretroviral-naïve, HIV-infected patients (median HIV-1 RNA 4.9 log_10 _copies/mL; CD4+ count 191 cells/mm^3^) randomly assigned to the FPV/r100 or ATV/r100 regimens.

**Results:**

At baseline, the FPV/r100 or ATV/r100 arms were well-matched for HIV-1 RNA (median, 4.9 log_10 _copies/mL [both]), CD4+ count (mean, 176 vs 205 cells/mm^3^). At week 48, intent-to-treat: missing/discontinuation = failure analysis showed similar responses to FPV/r100 and ATV/r100 (HIV-1 RNA < 50 copies/mL: 75% (40/53) vs 83% (44/53), p = 0.34 [Cochran-Mantel-Haenszel test]); mean CD4+ count change-from-baseline: +170 vs +183 cells/mm^3^, p = 0.398 [Wilcoxon rank sum test]). Fasting total/LDL/HDL-cholesterol changes-from-baseline were also similar, although week 48 median fasting triglycerides were higher with FPV/r100 (150 vs 131 mg/dL). FPV/r100-treated patients experienced fewer treatment-related grade 2–4 adverse events (15% vs 57%), with differences driven by ATV-related hyperbilirubinemia. Three patients discontinued TDF/FTC because their GFR decreased to <50 mL/min.

**Conclusion:**

The all-QD regimens of FPV/r100 and ATV/r100, plus TDF/FTC, provided similar virologic, CD4+ response, and fasting total/LDL/HDL-cholesterol changes through 48 weeks. Fewer FPV/r100-treated patients experienced treatment-related grade 2–4 adverse events.

## Background

The protease inhibitors fosamprenavir (FPV) and atazanavir (ATV) both have pharmacokinetic characteristics supporting their use once-daily (QD) boosted by small, subtherapeutic doses of ritonavir [1,2]. Mini-dose ritonavir inhibits CYP3A4 metabolism of APV (to which FPV is converted) and ATV, thereby decreasing their clearance, raising their plasma concentrations and exposure, and increasing their elimination half-lives [3]. To date, ritonavir 200 mg QD has been the recommended boosting dose for FPV QD regimens [4]. COL10053 showed that this dose provides a mean plasma APV concentration at the end of a dosing interval (C_τ_) of 1.4 μg/mL [5], which is over 9-fold above the mean APV protein binding-adjusted 50% inhibitory concentration (IC_50_) for wild-type virus (0.146 μg/mL) [6] and 4-fold above the historical C_τ _value observed with unboosted FPV 1400 mg BID (which, in turn, is 2-fold higher than the IC_50 _for wild-type virus) [4]. Ritonavir 100 mg QD is the only boosting dose recommended for use with ATV 300 mg [7]. This dose increases the ATV minimum plasma concentrations (C_min_) and area under the plasma concentration-time curve (AUC) 5-fold and 3-fold higher, respectively, than can be attained with unboosted ATV 400 mg QD [8].

As the incidence of gastrointestinal (GI) adverse events and unfavorable lipid elevations is directly proportional to the magnitude of ritonavir dose [3], using the lowest ritonavir dose possible for PI boosting would be expected to incur the fewest tolerability problems. With FPV, several pharmacokinetic studies that have evaluated a low ritonavir boosting dose of 100 mg QD reported that it provides a mean or median steady-state APV C_min _6- to 13-fold higher than the protein binding-corrected 50% inhibitory concentration (IC_50_) for wild-type HIV (0.146 μg/mL) [6], and that patients may experience better GI tolerability and less elevation in lipids [5,9-12].

As no study to date has compared the long-term efficacy of all-QD FPV/r100 and ATV/r100 regimens, we conducted a clinical trial evaluating their relative efficacy/safety in combination with QD tenofovir disoproxil fumarate (TDF)/emtricitabine (FTC) in antiretroviral-naïve, HIV-infected patients.

## Methods

### Patient selection

Male and non-pregnant female outpatients were eligible for enrollment if they were ≥ 18 years old, had HIV-1 infection documented by HIV-1 antibody enzyme-linked immunosorbent assay (ELISA) and Western blot test, were antiretroviral-naïve (<14 days of antiretroviral treatment), and were not receiving immunomodulatory drugs. Women were enrollable if they were postmenopausal, sterilized, or, if of childbearing potential, had a documented negative serum or urine pregnancy test (β-human chorionic gonadotropin) ≤ 7 days of study drug administration and used two methods of contraception (barrier method mandatory).

### Study design and treatment

This randomized, open-label, multicenter study was conducted between April 2005 and September 2006 at 16 outpatient sites in the United States. Enrollment was stratified at screening by plasma HIV-1 RNA to one of two strata (<100,000 and ≥ 100,000 copies/mL). To determine study eligibility, study candidates underwent a medical history, physical examination, CDC classification, viral load, CD4+ counts, clinical chemistry values, liver function tests, hematology, hepatitis B and C serology, and serum β-human chorionic gonadotropin test (women of childbearing age only) at the screening visit within 30 days pre-study. All enrolled patients were randomly assigned to one of two regimens for 48 weeks:

• FPV/r 1400 mg/100 mg QD + TDF 300 mg/FTC 200 mg QD

• ATV/r 300 mg/100 mg QD + TDF 300 mg/FTC 200 mg QD

FPV/r and TDF/FTC were administered with or without food and ATV and ritonavir were given together with food. The FPV dose was given as two 700-mg tablets of Lexiva^® ^(GlaxoSmithKline, Research Triangle Park, NC), TDF 300 mg/FTC 200 mg as one co-formulated tablet of Truvada^® ^(Gilead Sciences, Foster City, CA), ritonavir as one 100-mg soft-gel capsule of Norvir^® ^(Abbott Laboratories, North Chicago, IL), and ATV as two 150-mg capsules of Reyataz^® ^(Bristol-Myers Squibb, Princeton, NJ). Patients were counseled regarding adherence at weeks 0, 4, 12, 24, 36, and 48, and from the week 4 visit onward they were asked by study personnel about their level of adherence to each drug in their regimen.

If patients experienced FPV- or ATV-attributable (per investigator), treatment-limiting toxicities, they were discontinued from the study. If TDF/FTC-attributable, treatment-limiting toxicities occurred, abacavir (ABC) 600 mg/lamivudine (3TC) 300 mg (Epzicom^®^, GlaxoSmithKline) QD could be substituted. No other substitutions were allowed. All patients provided written informed consent to participate, and the protocol for the study was approved by the institutional review boards at each treatment site.

### Efficacy assessment

The primary efficacy measure was comparison of the proportion of patients with plasma HIV-1 RNA levels < 50 copies/mL at week 48, with secondary endpoints being proportion with HIV-1 RNA < 50 copies/mL at 24 weeks and < 400 copies/mL at 24 and 48 weeks; change from baseline in CD4+ counts at weeks 24 and 48; and HIV treatment-emergent resistance patterns (described in a separate paper).

HIV-1 RNA was measured, and change from baseline tabulated, at baseline (week 0), at weeks 4, 12, 24, 36 and 48, and at withdrawal using the Roche Amplicor MONITOR Ultrasensitive assay (version 1.5; lower limit of quantitation [LLOQ] 50 copies/mL) (Roche Diagnostics, Branchburg, New Jersey) and HIV-1 MONITOR Version 1.0 polymerase chain reaction (PCR) assay (LLOQ, 400 copies/mL) (Roche, Nutley, New Jersey). Virologic failure was defined two ways: 1) if prior to week 24, it was defined as a reduction of plasma HIV-1 RNA level to <50 copies/mL on two consecutive occasions with a subsequent increase to ≥ 400 copies/mL on two consecutive occasions 2–4 weeks apart; 2) if it occurred at week 24 or later, virologic failure was said to have occurred if plasma HIV-1 RNA level was ≥ 400 copies/mL on two consecutive occasions 2–4 weeks apart. Immunologic response was assessed by measuring change in CD4+ and CD8+ lymphocyte cell count from baseline by flow cytometry at weeks 0, 12, 24, 36, 48, and at withdrawal.

### Safety assessment

Patients were monitored for adverse events, laboratory abnormalities, and any HIV-related illnesses at weeks 0, 4, 12, 24, 36, and 48, and at withdrawal. The severity of adverse events was graded according to DAIDS criteria [13]. In addition, at weeks 0, 24, and 48, a fasting lipid panel was done and glomerular filtration rate (GFR) was estimated by the Modification of Diet in Renal Disease (MDRD) method [14]. In cases of elevated lipids, hypolipidemic agents could be prescribed at the discretion of the investigators. However, usage of lovastatin and simvastatin was prohibited, and atorvastatin and fluvastatin were to be used only on a precautionary basis in view of some potential for a drug interaction.

### Statistical analysis

A sample size of 50 patients per treatment arm was targeted based on practical rather than statistical considerations. No power calculations were made to determine this sample size. Analyses were performed on the intent-to-treat: exposed (ITT:E) population, which comprised all patients exposed to ≥ 1 dose of randomized study medication. Proportions of patients achieving < 50 copies/mL (primary efficacy parameter) and <400 copies/mL were analyzed by an ITT: observed analysis, which included all observed data, and an ITT: missing/discontinuation = failure (ITT: MD = F) analysis, in which patients with missing data or data collected after discontinuation of randomized study medication were considered failures. Between-treatment comparisons of these proportions were made by Cochran-Mantel-Haenszel test stratified by baseline HIV-1 RNA and differences in CD4+ count changes by Wilcoxon Rank-Sum test. Differences were considered statistically significant if *p *was < 0.05. Descriptive statistics alone were applied to all other data comparisons, including safety parameters.

## Results

### Patient characteristics and disposition

One hundred-six patients entered the study and 94 completed, 45 in the FPV/r100 arm and 49 in the ATV/r100 arm (Table 1). The baseline characteristics of patients in the two treatment arms were generally similar, except the FPV/r100 arm included more Caucasians and patients with a lower baseline CD4+ count. Most (84%) patients were male, median age was 40 years old, baseline median HIV-1 RNA was 4.9 log_10 _copies/mL (45% with ≥ 100,000 copies/mL), and median CD4+ count was 171 cells/mm^3^(Table 1). The population was ethnically diverse, with 40% African Americans, 23% of Hispanic ethnicity. Baseline MDRD-determined GFR was similar in the FPV/r100 and ATV/r100 arms (mean, 87.7 and 90.6 mL/min, respectively), but was 60–89 mL/min in 58% of patients in both the FPV/r100 arm (31/53) and ATV/r100 arm (31/53).

**Table 1:** Demographic characteristics (ITT exposed population)^a ^and disposition

	**FPV/r 1400/100 mg + TDF/FTC QD N = 53**	**ATV/r 300/100 mg + TDF/FTC N = 53**	**Total N = 106**
**Gender, n (%)**			
Male	42 (79%)	47 (89%)	89 (84%)
Female	11 (21%)	6 (11%)	17 (16%)
**Age, y**			
Median (range)	40 (22–64)	40 (20–58)	40 (20–64)
**Race, n (%)**^b^			
White	34 (64%)	26 (49%)	59 (56%)
Black	18 (34%)	24 (45%)	42 (40%)
Asian	0	1 (2%)	1 (<1%)
Other	1 (2%)	2 (4%)	2 (2%)
**HIV-1 RNA, log_10 _copies/mL**			
Median (range)	4.924 (2.775–6.320)	4.890 (3.167–6.362)	4.907 (2.775–6.362)
HIV-1 RNA < 100,000 copies/mL	29 (55%)	29 (55%)	58 (55%)
HIV-1 RNA ≥ 100,000 copies/mL	24 (45%)	24 (45%)	48 (45%)
**CD4+ cell count, cells/mm**^3^			
Median (range)	161 (19–524)	188 (19–488)	171 (19–524)
**CDC classification, n (%)**			
Class A (asymptomatic)	30 (57%)	34 (64%)	64 (60%)
Class B (symptomatic, non-AIDS)	14 (26%)	8 (15%)	22 (21%)
Class C (AIDS)	9 (17%)	11 (21%)	20 (19%)
**Mean GFR (by MDRD), mL/min/1.73 m**^2^	87.7 (± 20.4)	90.6 (± 18.0)	
**Study Withdrawals**			
Completed	45 (85%)	49 (92%)	94 (89%)
Prematurely withdrawn	8 (15%)	4 (8%)	12 (11%)
Reason for premature withdrawal			
Adverse event	1 (2%)	1 (2%)	2 (2%)
Lost to follow-up	2 (4%)	0	2 (2%)
Protocol violation	1 (2%)	0	1 (<1%)
Protocol-defined virologic failure	4 (8%)	3 (6%)	7 (7%)

^a^Comprised all patients exposed to ≥ 1 dose of randomized study medication.

^b^12 patients in the FPV/r arm and 12 in the ATV/r arm were of Hispanic/Latino ethnicity.

The 12 patients who discontinued treatment prematurely did so for similar reasons, the most common being protocol-defined virologic failure. Three patients discontinued TDF/FTC because their GFR decreased to <50 mL/min, and TDF/FTC was replaced by ABC/3TC. No patients were discontinued from the study for non-compliance.

### Efficacy

#### Virologic response

**Figure 1:**
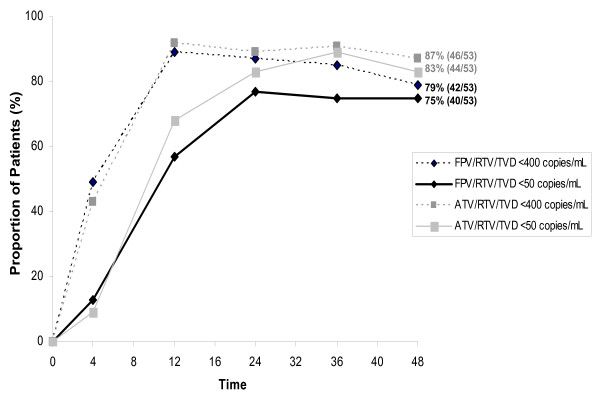
Proportion of patients with HIV-1 RNA < 400 copies/mL and < 50 copies/mL (intent-to-treat: missing/discontinuation = failure analysis).

Reduction in HIV-1 RNA was similarly rapid in the FPV/r100 and ATV/r100 arms, the median decrease from baseline in HIV-1 RNA at week 4 being 2.2 log_10 _copies/mL in each arm. Maximum reduction was seen at week 12 in both arms, and it remained undiminished through week 48. No significant differences (*p *> 0.05) were noted between the FPV/r100 and ATV/r100 regimens at week 48 (Figure 1) regarding proportion of patients achieving HIV-1 RNA < 50 copies/mL in the ITT: MD = F analysis (75% vs 83%) or ITT:observed analysis (89% vs 92%), nor in the proportion achieving < 400 copies/mL in these analyses (79% vs 87%); 93% vs 96%). Similarly, in patients with baseline HIV-1 RNA ≥ 100,000 copies/mL, week 48 results showed no differences between the FPV/r100 and ATV/r100 arms in proportion of patients achieving HIV-1 RNA < 50 copies/mL in the ITT: MD = F analysis (71% vs 75%) or ITT:observed analysis (85% vs 86%), nor in the proportion achieving < 400 copies/mL in these analyses (79% vs 79%; 95% vs 90%).

Virologic failure was observed in similar numbers of patients in the FPV/r100 and ATV/r100 arms (4 vs 3) despite the fact that pre-existing resistance to FPV or TDF/FTC, but not to ATV, was detected at baseline by genotype, phenotype, or both in 2 patients randomized to the FPV/r100 arm [15]. None of the 3 failures in the ATV/r100 arm had pre-existing resistance to ATV or TDF/FTC detected by population genotype or phenotype at baseline. A full delineation of resistance data is provided in a separate paper.

#### Immunologic response

**Figure 2:**
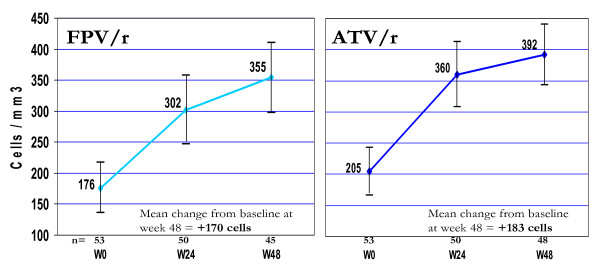
Mean CD4+ cell counts at all study visits.

CD4+ counts showed a similar pattern of increase over the course of the study in the FPV/r100 and ATV/r100 arms, with no statistically significant differences in magnitude of CD4+ count increase at any study visit (Figure 2). At week 48, the mean increase above baseline in CD4+ counts was 170 cells/mm^3 ^in the FPV/r100 arm and 183 cells/mm^3 ^in the APV/r100 arm (*p* = 0.398).

#### Safety

The overall incidence of *all *adverse events reported by at least 5% of patients, regardless of attributability to a particular treatment, is shown in Table 2. The most reported adverse events in FPV/r100-treated patients were diarrhea (53% [22/53]), nausea (13% [7/53]), fatigue (4% [2/53]), and headache (6% [3/53]). In the ATV/r100 arm, diarrhea (8 [15%]) and nausea (9% [5/53]) were reported less frequently and hyperbilirubinemia (43% [23/53]), ocular icterus (9% [5/53]), fatigue (8% [4/53]), and jaundice (6% [3/53]) more frequently (differences in frequency of adverse events not evaluated for statistical significance). As for treatment-related adverse events, most were grade 1 or 2 in severity. Evaluation of grade 2–4 treatment-related adverse events showed that these occurred more frequently in the ATV/r100 arm than in the FPV/r100 arm (57% vs 15%), driven largely by ATV-related hepatic effects. Grade 2–4 treatment-related GI adverse events reported in the FPV/r100 arm were diarrhea in 4 patients (8%) and nausea in 2 (4%). In the FPV/r100 arm, 1 patient had a grade 3 increased blood phosphorus and another had hypophosphatemia. Both patients were among the 3 whose GFR fell below 50 mL/min and who were withdrawn from the study (see below). Conversely, in the ATV/r100 arm, grade 3 treatment-related adverse events included increased blood bilirubin (26% [14/53]), increased aspartate transaminase (2% [1/53]), increased triglycerides (2% [1/53]), and hyperbilirubinemia (11%; 6/53]), and grade 4 treatment-related events included increased bilirubin (2% [1/53]) and increased alanine transaminase (2% [1/53]).

**Table 2:** All adverse events reported by frequency >5%

	**FPV/r 1400/100 mg + TDF/FTC QD N = 53**	**ATV/r 300/100 mg + TDF/FTC QD N = 53**
Diarrhea	28 (53%)	13 (25%)
Blood bilirubin increased	0	16 (30%)
Nausea	8 (15%)	6 (11%)
Rash	9 (17%)	5 (9%)
Fatigue	6 (11%)	7 (13%)
Headache	5 (9%)	3 (6%)
Hyperbilirubinemia	0	8 (15%)
Cough	4 (8%)	3 (6%)
Nasopharyngitis	3 (6%)	4 (8%)
Upper respiratory tract infection	2 (4%)	4 (8%)
Arthralgia	0	5 (9%)
Insomnia	2 (4%)	3 (6%)
Ocular icterus	0	5 (9%)
Syphilis	5 (9%)	0
Depression	3 (6%)	1 (2%)
Herpes zoster	1 (2%)	3 (6%)
Dizziness	3 (6%)	0
Jaundice	0	3 (6%)
Paresthesia	3 (6%)	0

**Figure 3:**
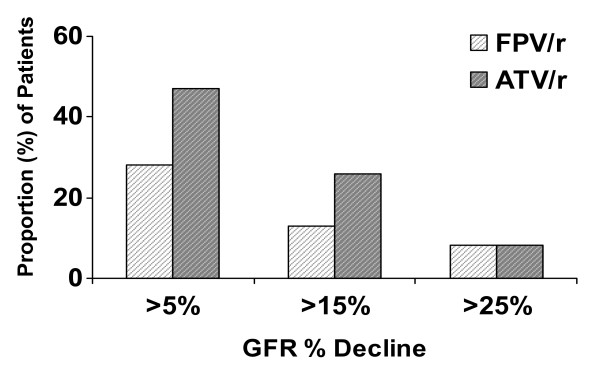
Proportion of patients with change in MDRD-determined glomerular filtration rate from baseline to week 48.

A similar proportion of patients in each arm experienced > 25% decrease in MDRD-determined GFR (Figure 3). Three patients on FPV/r100, but none on ATV/r100, discontinued TDF/FTC because their GFR decreased to <50 mL/min. At baseline, none of these patients had co-morbidities likely to account for reduction in GFR. During treatment, 2 of these 3 patients received no concurrent drugs known to adversely affect renal function, although the third began a 6-month course of diclofenac, a non-steroidal anti-inflammatory agent that has been implicated in rare reports of reduced creatinine clearance [16].

**Figure 4:**
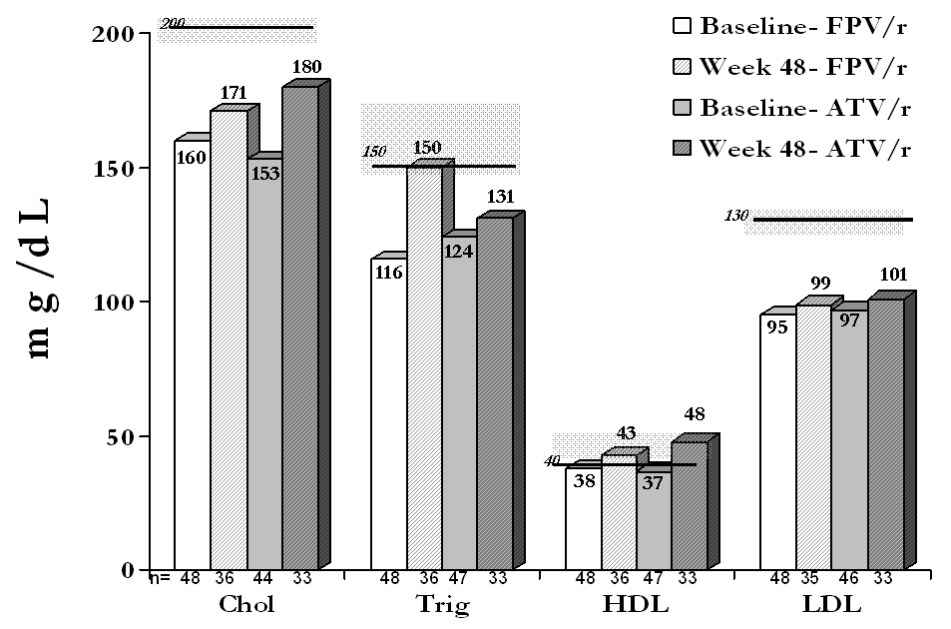
Median lipid values over the course of the study, with lines within shaded areas showing NCEP cut-offs.

Figure 4 presents median lipid values over the course of the study, with lines within shaded areas showing NCEP cut-offs [17]. FPV/r100 and ATV/r100 had comparable effects on median change from baseline at week 48 in total-cholesterol (+13 vs +10 mg/dL), LDL-cholesterol (+2 vs -6 mg/dL), and HDL-cholesterol (+11 vs +14 mg/dL). At week 48, a greater proportion of FPV/r100-treated patients (50% vs 39%) experienced an elevation in triglycerides that exceeded the NCEP normal range cut-off and, hence, were categorized as "borderline-high" or "high". Lipid-lowering agents were used by more patients in the FPV/r100 arm (*n *= 7) than in the ATV/r100 arm (*n *= 1). These agents included pravastatin (2), atorvastatin (2), cholestyramine (1), gemfibrozil (1), and nicotinic acid (1) in the FPV/r100 arm, and atorvastatin (1) in the ATV/r100 arm. Data contributed from patients after starting lipid-lowering agents were censored from the analysis.

## Discussion

In this study, the FPV/r100 and ATV/r100 arms performed similarly well with respect to virologic suppression and CD4+ cell enhancement. High virologic efficacy with the FPV/r100 regimen was expected based on the results of two other small clinical efficacy trials evaluating FPV/r100-containing regimens [18,19]. Hicks et al [18] reported that at 48 weeks, the proportion of ART-naïve patients (baseline median HIV-1 RNA 4.8 log_10 _copies/mL, CD4+ count 190 cells/mm^3^) able to achieve HIV-1 RNA levels < 50 copies/mL was as high or higher (depending on the type of analysis method), with an FPV/r100-containing QD regimen + ABC/3TC than with a FPV/r200-containing QD regimen with the same nucleoside backbone (79% vs 63% [ITT: M = F analysis], 92% vs 80% [observed analysis]). DeWit et al [19] evaluated FPV/r100 + TDF + 3TC (*n *= 57) (or FTC *n *= 19]) in ART-naïve patients (baseline median HIV-1 RNA 4.9 log_10 _copies/mL, CD4+ count 171 cells/mm^3^) and noted that at 48 weeks, 86% had HIV-1 RNA < 50 copies/mL (ITT: M = F) and CD4+ counts had increased above baseline by a median of 268 cells/mm^3^. TELEX II reported that patients stabilized (HIV-1 RNA < 50 copies/mL) for 48 weeks on FPV/r200 QD plus TDF/FTC 300/200 mg QD remained stabilized 4 weeks after reducing the ritonavir boosting dose to 100 mg QD [10].

The FPV/r100 regimen also is justified by four pharmacokinetic studies that reported little or no difference in the APV C_min _or AUC exposure in patients treated with FPV/r100 and FPV/r200 QD [5,9-11], possibly because ritonavir at 100 mg appears to predominantly inhibit CYP3A4 metabolism of APV, whereas ritonavir at 200 mg may have a combination of CYP3A4 inhibitory and induction effects [20]. As of October 12, 2007, ritonavir 100 mg QD boosting of FPV dosing was approved by the FDA [21] and listed as an alternative regimen in the DHHS HIV treatment guidelines [22]. Current International AIDS Society (IAS) treatment guidelines recommend ritonavir-boosted FPV as a recommended PI-based treatment for the initial treatment of HIV infection [23] and the British HIV Association (BHIVA) treatment guidelines list ritonavir-boosted FPV as an alternative first-line regimen [24].

The efficacy of the ATV/r100 regimen observed in our study was comparable to that reported in SHARE, which evaluated ATV/r100 + ABC/3TC in 111 ART-naïve patients (baseline median HIV-1 RNA 5.06 log_10 _copies/mL, CD4+ count 207 cells/mm^3^) [25]. At 48 weeks, 77% of ATV/r100-treated patients in SHARE achieved HIV-1 RNA < 50 copies/mL by ITT: M = F analysis and 90% by ITT: observed analysis, and their CD4+ cell count increased above baseline by a median of 188 cell/mm^3^. Inclusion of a 100-mg dose of ritonavir was important in the ATV regimen to counteract the previously documented TDF-related 23% reduction in ATV C_min _and 25% reduction in ATV exposure that is believed to be due to a physicochemical interaction of ATV and TDF in the intestine [26]. As ritonavir 100 mg increases ATV C_min _by 3-fold higher than is attainable with unboosted ATV 400 mg QD, this dose compensates for the negative pharmacokinetic effects of TDF [7]. As of January 2008, ATV/r100-based regimens are considered first-line PI regimens by DHHS HIV treatment guidelines [21], as recommended PI regimens by IAS guidelines [23], and as alternative PI regimens by BHIVA guidelines [24].

Grade 2–4 treatment-related adverse GI effects with FPV/r100 were observed, but the incidence was generally lower than has been reported with FPV boosted by r200 QD [5,18]. Thus, when a direct comparison of FPV/r100 vs FPV/r200 regimens was done in ART-naïve patients, the FPV/r100 regimen showed less grade 2–4 nausea (3% vs 5%) and diarrhea (14% vs 18%) [18]. Similarly, where such a comparison was made in healthy volunteers, a lower frequency of nausea (11% vs 27%) and loose stools (22% vs 29%) was also reported [5]. The high incidence of increased bilirubin in the ATV group was expected, as this has been described in previous ATV/r studies [27,28].

Fifty-eight percent of our patients entered the trial with GFR < 90 mL/min (31 in the ATV/r100 arm and 19 in the FPV/r100 arm), indicating some level of renal dysfunction pre-study in a substantial proportion of the study population. GFR changes were noted in both treatment arms, and reduction in GFR to below 50 mL/min resulted in 3 patients needing to be discontinued from the trial. Pharmacokinetic studies have established a drug-drug interaction between some PI's and TDF resulting in increased tenofovir concentrations [29-32]. Other data have suggested that diminished TDF renal tubule efflux is responsible for increased TDF concentrations within renal cells and plasma [33]. This finding has been postulated as a potential explanation for the decreased GFR seen in some patients treated with boosted PI's and TDF [34,35]. The boosted PIs ATV, lopinavir, saquinavir, and darunavir have been associated with an increase in tenofovir concentrations during co-administration [29-32], whereas this has not been observed with fosamprenavir (boosted and unboosted), indinavir (unboosted), tipranavir (boosted), and nelfinavir (unboosted) [31,36-38].

Wai et al [39] noted that the incidence of TDF-related GFR reduction is greater when RTV is administered concurrently in TDF-based regimens. This underscores the importance of achieving maximal boosting with the lowest possible RTV dose. As some factors that can contribute to renal decline in patients may not be known when they initially seek treatment, it is advisable that when a TDF/FTC backbone is being considered for use with PI-based therapy, renal function should be assessed at baseline and throughout treatment.

The magnitude of elevated total cholesterol, LDL-cholesterol, and especially triglycerides observed in the FPV/r100 arm was lower than has been reported with FPV/r 1400/200 mg QD regimens [12]. Median increase in HDL-cholesterol levels, a lipid change associated with reduction in cardiac risk, was observed in this study just as it has been in all other studies evaluating FPV/r100 [10,18]. A favorable change in lipid profile while maintaining clinical efficacy also has been reported within 4 weeks after ART-naïve patients were switched from FPV/r200 + TDF/FTC to FPV/r100 + TDF/FTC [10]. Where FPV/r100- and FPV/r200-containing regimens have been directly compared over 48 weeks, no major differences in lipid profiles were seen in one study (and no greater lipid effects at 48 weeks compared to 24 weeks) [18], whereas in the other study, FPV/r100 was associated with a less pronounced rise in triglycerides [5]. Although our study showed that the triglyceride increase at week 48 exceeded the NCEP cut-off in 50% of FPV/r100 vs 39% of ATV/r100 patients, they remained normal or just borderline high for most patients. In view of the minor lipid changes observed over 48 weeks with FPV/r 1400/100 mg QD, little or no lipid advantage was apparent for the ATV/r regimen.

This study is the first head-to-head clinical trial to compare FPV/r100- and ATV/r100-based regimens. The primary limitation of the study was its small sample size as it was done on a pilot basis. The study provides useful information since the study population was diverse with respect to gender, race, and ethnicity and mirrors the population where the epidemic is seen today.

## Conclusion

In conclusion, this pilot study showed that all-QD FPV/r100 and ATV/r100, in combination with TDF/FTC, provided similar virologic suppression and CD4+ cell increases through 48 weeks. A lower percentage of FPV/r100-treated patients experienced treatment-related grade 2–4 adverse events, and total/LDL/HDL cholesterol changes were generally similar.

## Competing interests

KYS has been a consultant to Bristol-Myers Squibb, GlaxoSmithKline, and Gilead Sciences, Inc.; WGW declares that he has no competing interests; ED has been a consultant and/or on the advisory board for Bristol-Myers Squibb, Gilead Sciences, Inc., GlaxoSmithKline, Roche Laboratories, Inc., and Vertex Pharmaceuticals, and has received grant and research support from Roche Laboratories, Inc. and Gilead Sciences, Inc.; MAF has served as an advisor for Progenics Pharmaceuticals, Inc. and Merck & Co., and has received research grants from Abbott Laboratories, Bristol-Myers Squibb, GlaxoSmithKline, and Progenics Pharmaceuticals, Inc.; QL, LLR, GEP, KAP, and CTL are employed by and own stock in GlaxoSmithKline.

## Authors' contributions

KYS was responsible for overall conduct and monitoring of safety parameters for the study. KYS, KAP, and CTL conceived the study design, which was reviewed, revised, and approved by all of the authors. CTL, KYS, KAP, QL, and GEP wrote, reviewed, and edited the protocol. GEP drafted the manuscript, which was reviewed and edited by all authors. KYS, WGW, ED, and MAF enrolled study subjects. CTL was responsible for logistical issues and study conduct, such as ensuring accurate completion of data collection forms. KYS, WGW, ED, MAF, GEP, KAP and CTL evaluated the clinical data from the study, LLR performed the virological analysis, and QL performed the statistical analysis. All authors read and approved the final manuscript.
